# Supplementary material for: Experimental cheat-sensitive quantum weak coin flipping
Source: Nat Commun. 2023 Apr 3;14:1855. doi: 10.1038/s41467-023-37566-x (PMC10070430; doi:10.1038/s41467-023-37566-x)
Supplement: Supplementary file 1 — Supplementary Information [file 41467_2023_37566_MOESM1_ESM.pdf]

Supplementary Information for "Experimental cheat-sensitive quantum weak coin flipping"

Simon Neves,[1] Verena Yacoub,[1] Ulysse Chabaud,[2,3] Mathieu Bozzio,[4] Iordanis Kerenidis,[5] and Eleni Diamanti[1]

[1]*Sorbonne Université, CNRS, LIP6, 4 Place Jussieu, Paris F-75005, France*
[2]*Institute for Quantum Information and Matter, California Institute of Technology, 1200 E California Blvd, Pasadena, CA 91125, USA*
[3]*DIENS, École Normale Supérieure, PSL University, CNRS, INRIA, 45 rue d'Ulm, Paris 75005, France*
[4]*University of Vienna, Faculty of Physics, Vienna Center for Quantum Science and Technology (VCQ), 1090 Vienna, Austria*
[5]*Université de Paris, CNRS, IRIF, 8 Place Aurélie Nemours, Paris 75013, France*

SUPPLEMENTARY NOTE 1: THEORETICAL PREDICTIONS

In this section we give some theoretical predictions for the results we observe in our experiments. In the first two subsections, we derive general expressions for event probabilities, for any values of beam splitter reflectivities x, y, and z. In the third subsection we obtain the values of these reflectivities which maximize fairness and correctness, when both parties are honest, as well as the probabilities of the different outcomes. In the fourth subsection, we show such predictions when one of the parties, Alice, is dishonest and performs an attack which we implement in this paper.

A. Photon propagation in the interferometer

We first describe the propagation of the photon in the interferometer (see Supplementary Figure 1), for any values of x, y, z, and deduce the probabilities of the different events. To simplify our proofs, we neglect dark counts and double-pair emissions. Experimental details in the following paragraphs support the legitimacy of this approximation. In this scenario, when Alice detects a photon in detector D_{herald}, then exactly one photon is generated, corresponding to the action of the creation operator $a_1^\dagger$. Some first losses occur when coupling the photon to single-mode fibers, such that the operator transforms as:

$$a_1^\dagger \longrightarrow \sqrt{\eta_c}\, a_1^\dagger, \tag{1}$$

with η_c being the induced transmission. Then Alice sends the photon to a BS of reflectivity x:

$$\sqrt{\eta_c}\, a_1^\dagger \longrightarrow \sqrt{x\eta_c}\, a_1^\dagger + \sqrt{(1-x)\eta_c}\, a_2^\dagger, \tag{2}$$

where 1 (resp. 2) stands for the reflected (resp. transmitted) mode. Alice keeps mode 1 and Bob gets mode 2. On each side, the photon undergoes losses due to fiber transmission and connectors, storage, and diverse other components. We note η_{A1} (resp. η_{B1}) the transmission on Alice's (resp. Bob's) side. Some phases are also induced by the propagation, and we note Φ_{A1} (resp. Φ_{B1}) the phase introduced on Alice's (resp. Bob's) side. In this way, we get the following transformation:

$$\sqrt{x\eta_c}\, a_1^\dagger + \sqrt{(1-x)\eta_c}\, a_2^\dagger \longrightarrow \sqrt{x\eta_c\eta_{A1}}\, e^{i\Phi_{A1}} a_1^\dagger + \sqrt{(1-x)\eta_c\eta_{B1}}\, e^{i\Phi_{B1}} a_2^\dagger. \tag{3}$$

Bob sends the photon to a BS of reflectivity y:

$$\begin{aligned}&\sqrt{x\eta_c\eta_{A1}}\, e^{i\Phi_{A1}} a_1^\dagger + \sqrt{(1-x)\eta_c\eta_{B1}}\, e^{i\Phi_{B1}} a_2^\dagger \\ &\qquad\longrightarrow \sqrt{x\eta_c\eta_{A1}}\, e^{i\Phi_{A1}} a_1^\dagger + \sqrt{(1-x)y\eta_c\eta_{B1}}\, e^{i\Phi_{B1}} a_2^\dagger + \sqrt{(1-x)(1-y)\eta_c\eta_{B1}}\, e^{i\Phi_{B1}} a_3^\dagger.\end{aligned} \tag{4}$$

Bob sends the third mode to the detector D_B, inducing another loss. We note η_y the transmission, including the detector efficiency, and we have $\eta_B^y = \eta_c\eta_{B1}\eta_y$ (here we omit the dephasing as no interference will occur on this mode). The second mode undergoes some loss and dephasing, and we note η_{B2} and Φ_{B2} the transmission and dephasing. There we note $\eta_B = \eta_c\eta_{B1}\eta_{B2}$ the total loss on Bob's arm of the interferometer, and $\Phi_B = \Phi_{B1} + \Phi_{B2}$ the total dephasing. On Alice's side, the path depends on the detection of the third mode that triggers the optical switch. In

absence of dark counts and when Bob is honest, a detection on the third mode means no detection will occur on Alice's verification detector, such that Bob is not sanctioned and wins the coin flip. In other words, Alice trusts Bob's measurement on the third mode, such that we can omit her verification detector and the optical switch. In that case she simply sends the first mode to Bob to proceed to verification of the state. That mode undergoes some loss and dephasing, and we note η_{A2} and Φ_{A2} the transmission and dephasing. There we note $\eta_A = \eta_c \eta_{A1} \eta_{A2}$ the total loss on Alice's arm of the interferometer, and $\Phi_A = \Phi_{A1} + \Phi_{A2}$ the total dephasing. The total transformation becomes:

$$\begin{aligned}\sqrt{x\eta_c\eta_{A1}}\, e^{i\Phi_{A1}} a_1^\dagger + \sqrt{(1-x)y\eta_c\eta_{B1}}\, e^{i\Phi_{B1}} a_2^\dagger + \sqrt{(1-x)(1-y)\eta_c\eta_{B1}}\, e^{i\Phi_{B1}} a_3^\dagger \\ \longrightarrow \sqrt{x\eta_A}\, e^{i\Phi_A} a_1^\dagger + \sqrt{(1-x)y\eta_B}\, e^{i\Phi_B} a_2^\dagger + \sqrt{(1-x)(1-y)\eta_B^y}\, e^{i\Phi_B} a_3^\dagger.\end{aligned} \tag{5}$$

After receving the first mode, Bob makes it interfere with the second mode on a BS of reflectivity z, such that we get:

$$\begin{aligned}&\sqrt{x\eta_A}\, e^{i\Phi_A} a_1^\dagger + \sqrt{(1-x)y\eta_B}\, e^{i\Phi_B} a_2^\dagger + \sqrt{(1-x)(1-y)\eta_B^y}\, e^{i\Phi_B} a_3^\dagger \\ &\longrightarrow \left(\sqrt{xz\eta_A}\, e^{i\Phi_A} + \sqrt{(1-x)y(1-z)\eta_B}\, e^{i\Phi_B}\right) a_1^\dagger \\ &\quad - \left(\sqrt{x(1-z)\eta_A}\, e^{i\Phi_A} - \sqrt{(1-x)yz\eta_B}\, e^{i\Phi_B}\right) a_2^\dagger + \sqrt{(1-x)(1-y)\eta_B^y}\, e^{i\Phi_B} a_3^\dagger.\end{aligned} \tag{6}$$

Bob sends the first and second modes to detectors D_{V_1} and D_{V_2}, with efficiencies η_{V_1} and η_{V_2}, and we note $\eta_A^{V_1} = \eta_A\,\eta_{V_1}$, $\eta_A^{V_2} = \eta_A\,\eta_{V_2}$, $\eta_B^{V_1} = \eta_B\,\eta_{V_1}$, and $\eta_B^{V_2} = \eta_B\,\eta_{V_2}$. Up to an irrelevant global phase $e^{i\Phi_A}$, we get:

$$\begin{aligned}&\left(\sqrt{xz\eta_A}\, e^{i\Phi_A} + \sqrt{(1-x)y(1-z)\eta_B}\, e^{i\Phi_B}\right) a_1^\dagger \\ &\quad - \left(\sqrt{x(1-z)\eta_A}\, e^{i\Phi_A} - \sqrt{(1-x)yz\eta_B}\, e^{i\Phi_B}\right) a_2^\dagger + \sqrt{(1-x)(1-y)\eta_B^y}\, e^{i\Phi_B} a_3^\dagger \\ &\longrightarrow \left(\sqrt{xz\eta_A^{V_1}} + \sqrt{(1-x)y(1-z)\eta_B^{V_1}}\, e^{i\Delta\Phi}\right) a_1^\dagger \\ &\quad - \left(\sqrt{x(1-z)\eta_A^{V_2}} - \sqrt{(1-x)yz\eta_B^{V_2}}\, e^{i\Delta\Phi}\right) a_2^\dagger + \sqrt{(1-x)(1-y)\eta_B^y}\, e^{i\Delta\Phi} a_3^\dagger,\end{aligned} \tag{7}$$

where $\Delta\Phi = \Phi_B - \Phi_A$ is the phase difference. We deduce the detection probabilities in each detector:

$$P_{V_1} = \mathbb{P}_h\big((b, v_1, v_2) = (0,1,0)\big) = xz\eta_A^{V_1} + (1-x)y(1-z)\eta_B^{V_1} + 2\cos(\Delta\Phi)\sqrt{x(1-x)yz(1-z)\eta_A^{V_1}\eta_B^{V_1}}, \tag{8}$$

$$P_{V_2} = \mathbb{P}_h\big((b, v_2) = (0,1)\big) = x(1-z)\eta_A^{V_2} + (1-x)yz\eta_B^{V_2} - 2\cos(\Delta\Phi)\sqrt{x(1-x)yz(1-z)\eta_A^{V_2}\eta_B^{V_2}}, \tag{9}$$

$$P_{D_B} = \mathbb{P}_h\big((b, a) = (1,0)\big) = (1-x)(1-y)\eta_B^y. \tag{10}$$

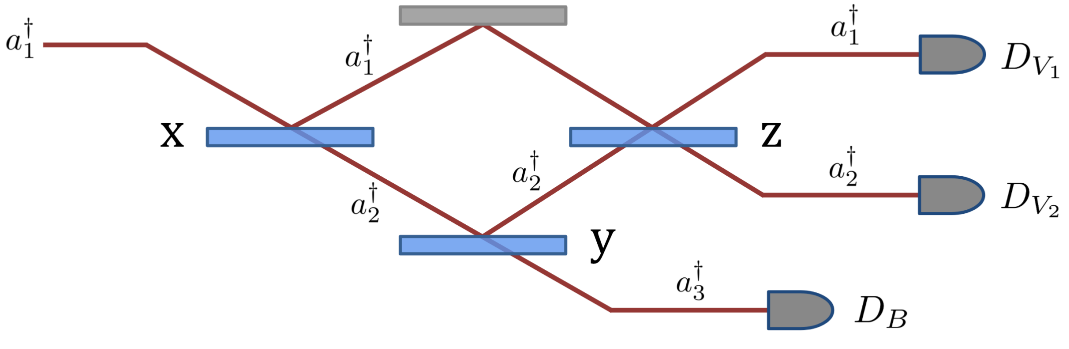

Supplementary Figure 1. Sketch of the interferometer with most relevant notations.

B. Phase fluctuations

In our experiment, the phase difference $\Delta\Phi$ evolves through time, because of thermal fluctuations and diverse vibrations or noise. Slow phase drifts, typically caused by thermal fluctuations, are generally resolved when counting

photons, provided the photon rate is high enough. Fast phase fluctuations however, typically caused by noise, are hard to resolve by counting photons, due to low rates and detector recovery time. Hence, the probabilities P_{V_1} and P_{V_2} are averaged over the typical temporal resolution τ of our detectors. We distinguish two types of behaviour in the phase difference $\Delta\Phi(t) = \Delta\Phi_f(t) + \Delta\Phi_s(t)$, with $\Delta\Phi_f(t)$ corresponding to fast fluctuations of typical period $\tau_f \ll \tau$, and $\Delta\Phi_s(t)$ corresponding to slow fluctuations of typical period $\tau_s \gg \tau$. For fast fluctuations, the average value $\langle\cos\Delta\Phi_f\rangle_\tau$ is approximately constant. For slow fluctuations, the value of $\Delta\Phi_s(t)$ is approximately constant over a time lapse of τ. In this way, we get:

$$\begin{aligned}\langle\cos\Delta\Phi\rangle_\tau(t) &= \langle\cos(\Delta\Phi_f + \Delta\Phi_s)\rangle_\tau(t)\\ &= \langle\cos\Delta\Phi_f\cos\Delta\Phi_s\rangle_\tau(t) - \langle\sin\Delta\Phi_f\sin\Delta\Phi_s\rangle_\tau(t_0)\\ &= \langle\cos\Delta\Phi_f\rangle_\tau\cos\Delta\Phi_s(t) - \langle\sin\Delta\Phi_f\rangle_\tau\sin\Delta\Phi_s(t)\\ &= v\,\big(C\cdot\cos\Delta\Phi_s(t) - S\cdot\sin\Delta\Phi_s(t)\big),\end{aligned} \tag{11}$$

with $v := \sqrt{\langle\cos\Delta\Phi_f\rangle_\tau^2 + \langle\sin\Delta\Phi_f\rangle_\tau^2}$, $C := \langle\cos\Delta\Phi_f\rangle_\tau/v$, and $S := \langle\sin\Delta\Phi_f\rangle_\tau/v$. By definition we have $C^2+S^2 = 1$, so there exists a phase ϕ_{eff} with $C = \cos\phi_{\text{eff}}$ and $S = \sin\phi_{\text{eff}}$. We then get:

$$\begin{aligned}\langle\cos\Delta\Phi\rangle_\tau(t) &= v\,\big(\cos\phi_{\text{eff}}\cos\Delta\Phi_s(t) - \sin\phi_{\text{eff}}\sin\Delta\Phi_s(t)\big)\\ &= v\,\cos(\Delta\Phi_s(t) + \phi_{\text{eff}}).\end{aligned} \tag{12}$$

Here ϕ_{eff} appears as an additional constant dephasing, such that we can include it inside the slow dephasing $\Delta\Phi_s(t_0)$. Effectively, it means taking $\phi_{\text{eff}} = 0$, such that $S = 0$ and $\langle\sin\Delta\Phi_f\rangle_\tau = 0$. In this way, we have:

$$\langle\cos\Delta\Phi\rangle_\tau(t) = v\,\cos\Delta\Phi_s(t) \tag{13}$$

with $v = |\langle\cos\Delta\Phi_f\rangle_\tau| \in [0,1]$, that we later interpret as the interference visibility. Now we average P_{V_1} and P_{V_2}:

$$\langle P_{V_1}\rangle_\tau(t) = xz\eta_A^{V_1} + (1-x)y(1-z)\eta_B^{V_1} + 2v\cos\big(\Delta\Phi_s(t)\big)\sqrt{x(1-x)yz(1-z)\eta_A^{V_1}\eta_B^{V_1}}, \tag{14}$$

$$\langle P_{V_2}\rangle_\tau(t) = x(1-z)\eta_A^{V_2} + (1-x)yz\eta_B^{V_2} - 2v\cos\big(\Delta\Phi_s(t)\big)\sqrt{x(1-x)yz(1-z)\eta_A^{V_2}\eta_B^{V_2}}, \tag{15}$$

which are the effective expressions of P_{V_1} and P_{V_2} we can use for our estimations in the following. For this reason, we omit the averaging and time dependence in the remainder of the paper.

C. Predictions with honest parties

We now consider a protocol where both parties are honest, and we derive the parameters x, y and z that maximize the fairness and correctness. The fairness condition imposes:

$$\mathbb{P}_h\big((b,a) = (1,0)\big) = \mathbb{P}_h\big((b,v_1,v_2) = (0,1,0)\big), \tag{16}$$

and the correctness condition imposes:

$$\mathbb{P}_h\big((b,a) = (1,1)\big) = \mathbb{P}_h\big((b,v_2) = (0,1)\big) = 0. \tag{17}$$

As we neglected dark counts and double-pair emissions, we already have $\mathbb{P}_h\big((b,a) = (1,1)\big) = 0$. However, we have *a priori* $\mathbb{P}_h\big((b,v_2) = (0,1)\big) > 0$ for any non-trivial parameters $x,y,z \notin \{0,1\}$ (these cases do not allow to verify the fairness condition). It is therefore impossible in principle to verify the correctness condition. Still, we minimize $\mathbb{P}_h\big((b,v_2) = (0,1)\big)$ in order to approach the condition. As a reminder, we have:

$$\mathbb{P}_h\big((b,v_2) = (0,1)\big) = P_{V_2} = x(1-z)\eta_A^{V_2} + (1-x)yz\eta_B^{V_2} - 2v\cos\big(\Delta\Phi_s\big)\sqrt{x(1-x)yz(1-z)\eta_A^{V_2}\eta_B^{V_2}}. \tag{18}$$

We first notice that minimizing that expression imposes $\Delta\Phi_s = 0$. Now we recall that $\eta_A^{V_2} = \eta_A\eta_{V_2}$ and $\eta_B^{V_2} = \eta_B\eta_{V_2}$, and define $\Pi_A = x\eta_A$ and $\Pi_B = (1-x)y\eta_B$ that we interpret as the probabilities of measuring the photon in Alice's side or Bob's side, before the last tunable BS. We can then rewrite the probability:

$$P_{V_2} = \eta_{V_2}\cdot\left((1-z)\Pi_A + z\Pi_B - 2v\sqrt{z(1-z)\Pi_A\Pi_B}\right). \tag{19}$$

We can then define a variable $\xi := \frac{\Pi_A}{\Pi_{\text{tot}}} \in]0,1[$ with $\Pi_{\text{tot}} = \Pi_A + \Pi_B$, such that:

$$P_{V_2} = \eta_{V_2}\Pi_{\text{tot}}\left((1-z)\xi + z(1-\xi) - 2v\sqrt{z(1-z)\xi(1-\xi)}\right). \tag{20}$$

P_{V_2} is minimized for $\partial P_{V_2}/\partial\xi = 0$ and $\partial P_{V_2}/\partial z = 0$. One can easily show that for $v < 1$, this system has a single solution $\xi = z = 1/2$, such that $\Pi_A = \Pi_B$. This drastically simplifies the expressions of the probabilities:

$$P_{V_1} = x\eta_A^{V_1}(1+v), \tag{21}$$
$$P_{V_2} = x\eta_A^{V_2}(1-v), \tag{22}$$
$$x\eta_A^{V_1} = (1-x)y\eta_B^{V_1}. \tag{23}$$

The case $v = 1$ corresponds to a perfect interference, and implies $\partial P_{V_2}/\partial\xi = \partial P_{V_2}/\partial z$ for any set of parameters ξ and z. This way, an infinite number of ξ and z satisfy $\partial P_{V_2}/\partial\xi = 0$ and $\partial P_{V_2}/\partial z = 0$. One can therefore impose another condition, such as the *balance* condition introduced in [1], in order to find a unique solution (ξ, z). This case is not relevant for our study as the interference is imperfect as in all practical scenarios.

Now we can apply the fairness condition, which in the honest case with no dark counts and no double-pair emission reduces to $P_{V_1} = P_{D_B}$. This gives the following equation on the parameters:

$$x\eta_A^{V_1}(1+v) = (1-x)(1-y)\eta_B^{y}. \tag{24}$$

Combining Eqs. (23) and (24), we can derive the expressions of the three parameters x, y and z that optimize both fairness and correctness:

$$x_h = \left[1 + \frac{\eta_A^{V_1}}{\eta_B^{V_1}} + \frac{\eta_A^{V_1}}{\eta_B^{y}}(1+v)\right]^{-1}, \tag{25}$$

$$y_h = \left[1 + \frac{\eta_B^{V_1}}{\eta_B^{y}}(1+v)\right]^{-1}, \tag{26}$$

$$z_h = \frac{1}{2}. \tag{27}$$

Then, the probabilities of the different events are calculated straightforwardly:

$$\mathbb{P}_h\big(\text{Alice wins}\big) = \mathbb{P}_h\big(\text{Bob wins}\big) = P_{V_1} = P_{D_B} = x_h\eta_A^{V_1}(1+v), \tag{28}$$
$$\mathbb{P}_h\big(\text{Bob sanctioned}\big) = 0, \tag{29}$$
$$\mathbb{P}_h\big(\text{Alice sanctioned}\big) = P_{V_2} = x_h\eta_A^{V_2}(1-v). \tag{30}$$

This confirms that the correctness condition is not fulfilled in general, but is approached when v gets close to 1, i.e. when the noise is low enough. We detail the experimental procedure for that noise cancellation in a later section. One can also notice that the condition $\Pi_A = \Pi_B$, which later translates to Eq. (23), gives the expected result that the two arms of the interferometer should have equal power in order to display an optimized interference. This should be kept in mind when experimentally setting up the parameters. Finally, by keeping the same reflectivities, and comparing the values of P_{V_1} and P_{V_2} when $\Delta\Phi_s = 0$ or $\Delta\Phi_s = \pi$, we get:

$$v = \left|\frac{P_{V_1}(\Delta\Phi_s = 0) - P_{V_1}(\Delta\Phi_s = \pi)}{P_{V_1}(\Delta\Phi_s = 0) + P_{V_1}(\Delta\Phi_s = \pi)}\right| = \left|\frac{P_{V_2}(\Delta\Phi_s = 0) - P_{V_2}(\Delta\Phi_s = \pi)}{P_{V_2}(\Delta\Phi_s = 0) + P_{V_2}(\Delta\Phi_s = \pi)}\right|, \tag{31}$$

so we can indeed interpret v as the interference visibility, which can be easily evaluated experimentally. Finally, we mention that each path's transmission efficiency can be measured by setting the reflectivities and switch's state to trivial values $x, y, z, s \in \{0, 1\}$ given in Main Text Table 1, in which we also give the experimentally measured values of these efficiencies. From these efficiencies we can compute the above theoretically predicted reflectivities x_h, y_h and z_h, which maximize the fairness $\mathcal{F}$ and correctness $\mathcal{C}$. The evolution of these values with the communication distance are shown in a later section, in Supplementary Figure 4, together with the reflectivities measured in our experiments.

D. Predictions for a dishonest Alice

Now we derive results for the case when Alice is dishonest and Bob is honest. In general, Alice might be able to perform more sophisticated strategies, involving more complex quantum states, such as those mentioned in [1]. Yet, finding optimal cheating strategies for Alice in a practical scenario remains an open question. On the other hand, it was shown in [1] that the highest winning probability of a cheating Alice is bounded by her highest probability to win in the ideal case, i.e., when all experimental components (fibers, switch, detectors, etc.) are perfect. This bound is given by:

$$\mathbb{P}(\text{A. wins}) \leq 1 - (1 - y_h)(1 - z_h) \tag{32}$$

$$= \frac{1 + \frac{1}{2}\frac{\eta_B^{V_1}}{\eta_B^y}(1+v)}{1 + \frac{\eta_B^{V_1}}{\eta_B^y}(1+v)}, \tag{33}$$

where the second line is obtained using the expression of y_h and z_h from Eqs. (26) and (27). Note that while this bound is independent of the experimental parameters in [1], in our case these parameters appear since the values of y_h and z_h are fine-tuned to ensure the fairness condition. This bound is obtained by showing that, up to introducing additional losses in the last two modes of the setup which can only increase Alice's winning probability, the losses of the setup can be commuted through the interferometer to the input state prepared by Alice. Then, the security analysis is equivalent to that of the ideal protocol, up to the fact that Alice is restricted to lossy state preparation, which can only decrease her winning probability. We refer the reader to Appendix E of [1] for a detailed derivation.

To illustrate the cheat sensitivity of our protocol, we consider a naive strategy, by simply setting up a reflectivity $x > x_h$, which *a priori* favors Alice (such a strategy is optimal in the ideal case of a lossless protocol [1]). As Bob is honest, we still keep $y = y_h$ and $z = z_h = 1/2$ from Eqs. (6) and (7), and Alice's verification setup is not required. In that case we can derive the expressions for the probabilities of the different events:

$$\mathbb{P}(\text{A. wins}) = \langle P_{V_1} \rangle = \frac{1}{2}\left(x\eta_A^{V_1} + (1-x)y_h\eta_B^{V_1} + 2v\sqrt{x(1-x)y_h\eta_A^{V_1}\eta_B^{V_1}} \right), \tag{34}$$

$$\mathbb{P}(\text{A. sanctioned}) = \langle P_{V_2} \rangle = \frac{1}{2}\left(x\eta_A^{V_2} + (1-x)y_h\eta_B^{V_2} - 2v\sqrt{x(1-x)y_h\eta_A^{V_2}\eta_B^{V_2}} \right), \tag{35}$$

$$\mathbb{P}(\text{B. wins}) = P_{D_B} = (1-x)(1-y_h)\eta_B^y. \tag{36}$$

These come straightforwardly from Eqs. (10), (14) and (15), by noting that a dishonest Alice would still set $\Delta\Phi_s = 0$, which maximizes her winning probability and minimizes her sanction probability. This gives the curves plotted in Fig. 5(a) in the main text.

E. Case of two dishonest parties

Most quantum two-party computation security models do not consider both parties being dishonest at the same time, since security makes sense from the perspective of an honest party willing to protect against a malicious adversary. This threat model is still very general however, as one does not make any assumption on which of the two parties is dishonest: the protocol is therefore always secure for both an honest Alice and an honest Bob. In the case of our protocol however, understanding the double-dishonest scenario is fairly straightforward, and in fact reduces to a fully classical protocol. Since the protocol is designed in such a way that the same party (Bob) always declares the outcome of the flip first (while the verification is then performed by the losing party), Bob cannot win in any other way than declaring himself as the winner. The best that Alice can do is to then stop Bob from winning, claiming that she caught him cheating. Thus, the protocol will always abort, which is a desirable outcome in such a dishonest scenario. The case only becomes a little more complex when one considers sanctioning dishonest aborts. In that case, Bob will always be sanctioned for cheating first, even though Alice was also dishonest.

SUPPLEMENTARY NOTE 2: EXPERIMENTAL DETAILS

A. Heralded single-photon source

The heralded single photons are emitted via type-II spontaneous parametric down-conversion (SPDC) in a ppKTP crystal. The energy conservation and quasi-phase matching imposes the following relations between the pump, signal and idler frequencies and spatial momenta:

$$\Delta\omega = \omega_p - \omega_s - \omega_i = 0 \tag{37}$$

$$\Delta k = k_p - k_s - k_i - \frac{2\pi}{\Lambda} = 0, \tag{38}$$

where ω_p, ω_s, and ω_i are the pump, signal and idler frequencies, k_p, k_s, and k_i are their spatial momenta and Λ is the crystal poling period. The pump laser center wavelength is $\lambda_p = 770\,\mathrm{nm}$, and the poling period is $\Lambda = 46.2\,\mu\mathrm{m}$. With these parameters, at room temperature, using Sellmeier's equations from [2, 3] for ppKTP's optical indices $n_p = 1.76$, $n_s = 1.73$, and $n_i = 1.82$, the signal and idler photon wavelengths are around $\lambda_s \simeq 1541.5\,\mathrm{nm}$ and $\lambda_i \simeq 1538.5\,\mathrm{nm}$.

The pump focus and spectral bandwidth, as well as the crystal length, are of particular relevance to determine or optimize key properties of the photons, such as their coherence length or the heralding efficiency. The pump is focused on the middle of our $L = 30\,\mathrm{mm}$-long crystal, with a waist of $w_p \simeq 315\,\mu\mathrm{m}$. The signal photon's coupling mode has a waist $w_s \simeq 190\,\mu\mathrm{m}$, and the idler photon's is $w_i \simeq 218\,\mu\mathrm{m}$. In this way, we get the focusing parameters $\xi_p = \frac{\pi w_p^2 n_p}{\lambda_p L} \simeq 24$ for the pump, $\xi_s = \frac{\pi w_s^2 n_s}{\lambda_s L} \simeq 4.2$ for the signal, and $\xi_i = \frac{\pi w_i^2 n_i}{\lambda_i L} \simeq 5.9$ for the idler. We see that the pump beam, as well as photon modes, are close to collimated on the crystal scale. Under these conditions we can consider the spatial state to be uncorrelated from the spectral state [4, 5]. The spectral state of the photons is given by the following expression:

$$|\psi_{si}\rangle = \frac{1}{\mathcal{N}} \iint d\omega_s d\omega_i \gamma(\omega_i, \omega_s) a^\dagger_{\omega_s} a^\dagger_{\omega_i} |0_s, 0_i\rangle\,, \tag{39}$$

where $\mathcal{N}$ is a normalization factor, and $\gamma(\omega_i, \omega_s)$ is the so-called *joint spectral amplitude* (JSA), which takes the form:

$$\gamma(\omega_i, \omega_s) = \alpha(\omega_i + \omega_s) \cdot \phi(\omega_i, \omega_s) \tag{40}$$

where $\alpha(\omega) = \exp\bigl(-\frac{(\omega-\omega_p)^2}{2\sigma_p^2}\bigr)$ is the pump spectrum with ω_p the central frequency and σ_p the bandwidth, $\phi(\omega_s, \omega_i) = \mathrm{sinc}(\Delta k/L)$ is the phase matching amplitude. From the JSA we can extract the Schmidt number K of the pair, and the purity P of each of the photons using $P = 1/K$. Considering the properties of our crystal and our pump Laser's bandwidth $\sigma_p = 0.2\,\mathrm{nm}$, we expect a purity $P \simeq 0.85$, with a JSA shown in Supplementary Figure 2. Under these conditions, the spectral state is close to pure such that we can evaluate the spectral FWHM $1\,\mathrm{nm}$ of the single photons as well as their coherence length $\simeq 2.4\,\mathrm{mm}$.

B. Error management

Different factors can generate undesired detection events in our protocol, therefore triggering outcomes that would otherwise be of low probability. This is true in particular for sanction outcomes, triggered by a detection in detector D_A or D_{V_2} which should never happen when a party is honest. Thus managing these error sources is of major importance in order to satisfy the correctness condition in particular, but also to minimize undesired outcomes in general. Most of these outcomes arise from Bob's verification procedure, which relies on a Mach–Zehnder interferometer. If this interference is of poor visibility, then D_{V_2} can be triggered even if Alice is being honest, and her winning probability is also substantially lowered. Considering the length of this interferometer ($\geq 300\,\mathrm{m}$), the visibility is limited by two main factors, namely the coherence length and phase fluctuations.

The coherence length of photons is $\simeq 2.4\,\mathrm{mm}$, which is small enough to start losing coherence after a few hours of experiments. This is mostly caused by length variations in the interferometer arms due to thermal fluctuations ($\simeq 2.4\,\mathrm{mm/°C}$ for a $300\,\mathrm{m}$ arm). We therefore regularly fine tune the length of one arm of the interferometer, using a free-space micro-metric delay line.

In first approximation, phase fluctuations can be separated into two regimes. Slow phase fluctuations, of typical frequency $\lesssim 1\,\mathrm{Hz}$, are again caused by thermal variations. We can easily measure them, and then either correct them

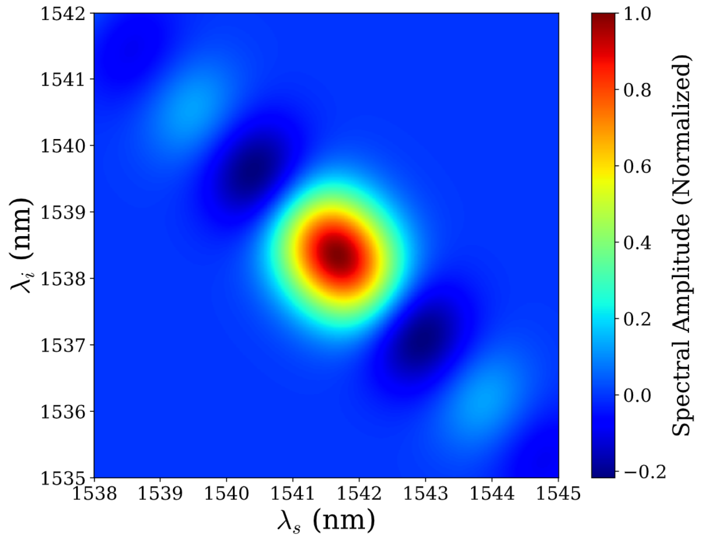

Supplementary Figure 2. Simulated joint spectral amplitude of the photon pairs emitted in our experiment.

or simply post-select the desired phase differences. We adopt the latter method in our experiment, which does not threaten the security of the protocol, as parties are allowed to monitor the phase in real time and make the protocol start only when it is set at $\Delta\Phi_s = 0$. Fast phase fluctuations, however, are caused by noise spanning the audible spectrum from 20 Hz to 2 kHz. This noise is amplified by the 300 m fiber spools, which act as sort of microphone. These fluctuations are hard to resolve with our single-photon rate of a few 10 kHz, such that the interference pattern is averaged on that noise, and we witness an interference visibility of approximately $v \simeq 80\%$. In order to characterize that noise, we measure the interference pattern with a continuous diode laser and a fast photodiode (see Supplementary Figure 3). Without any sound insulation, the noise in the interference fluctuation spans the audible spectrum with a power spectral density of approximately $\simeq 7 \times 10^{-3}\,\mathrm{V}^2/\mathrm{Hz}$. In order to mitigate this effect, we wrap the fiber spools into several layers of sound-absorbing floating parquet underlay. The power spectral density then drops to less than $1 \times 10^{-3}\,\mathrm{V}^2/\mathrm{Hz}$ except for some specific frequencies. The total noise power is divided by a factor greater than $\gtrsim 11$. The measured visibility then reaches $v \gtrsim 96\%$.

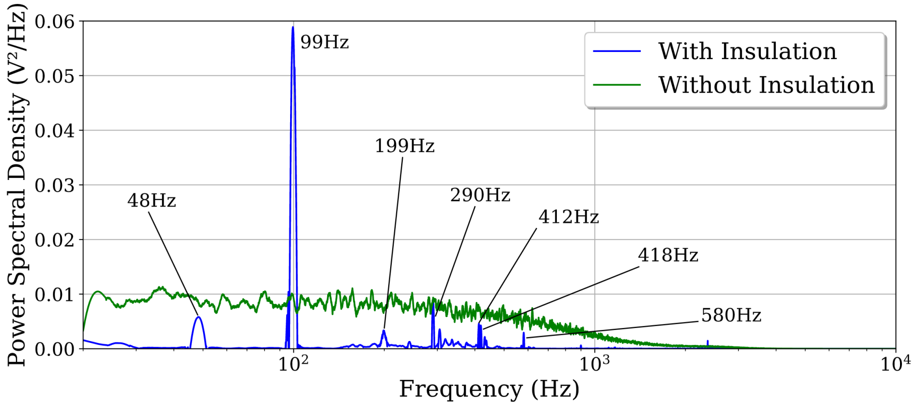

Supplementary Figure 3. Noise spectrum measured in the interferometer, using a continuous laser and a fast photodiode, with and without sound insulation on the fiber spools. When adding the insulation, the noise is low enough to distinguish peaks coming from the main sources of sound in the lab: 48 Hz, 99 Hz, and 199 Hz are emitted by the compressor plugged to the detectors cryostat, 290 Hz and 580 Hz are emitted by the pump in the cold water circuit, which cools down the compressor, and 412 Hz and 418 Hz are emitted by the laser chiller.

Undesired outcomes can also be triggered by double-pair emission inside the crystal, and dark counts in the

detectors. The double-pair emission rate is easily minimized by lowering the pump power, such that the probability p of producing a photon pair in a pump pulse is lower than 0.1. In this way, the probability of generating two pairs in the same pulse is $p^2 \ll p$, such that double-pair emission becomes negligible. In our experiment, we evaluate $p \simeq 0.015$. Dark counts rates are made particularly low by detecting the heralded single photon with SNSPDs with $< 100\,\text{Hz}$-dark count rate, as well as $< 50\,\text{ps}$-timing jitter electronics. We use the pump internal signal in order to synchronize a 500 ps detection gate with each of the detectors signal. Furthermore, all signal-photon detections are conditioned on a heralding photon detection. The probability of detecting a dark count during a protocol run is then $5 \cdot 10^{-8}$, such that undesired outcomes due to dark counts are negligible. However, we still use an APD, with substantially higher dark count rate than SNSPDs, in order to detect the heralding photon. Such dark counts trigger protocol runs while no photon was emitted. This results in a slight increase of the abort probability, as the other detectors will not click in such a situation. We evaluate the rate of such runs to be $\lesssim 40\,\text{Hz}$, thanks to the gating applied by the pump laser signal. This way the surplus of abort probability caused by dark count is about $8 \cdot 10^{-4}$, which is negligible compared to the typical > 0.7 abort probability.

C. Optical switch and decision

During the decision step of the protocol, Bob's detection determines which party is winning, and which one is performing the verification. In our experiment, this decision is effectively taken into account by Alice via her optical switch. Hence, if Bob does not claim victory, the switch is in state "0" in order to send Alice's state to Bob, who performs the verification. If Bob claims victory, the switch goes to state "1" such that Alice keeps her state and performs the verification. In practice, we send the electronic signal from Bob's detector, together with the heralding signal, to a fast programmable logic AND gate, integrated in a time controller. This AND gate filters out potential detection events outside of the protocol, which might saturate the optical switch. The gate's output signal is then sent to the optical switch, which executes the decision.

Two timings must be set carefully in order to send the photon in the appropriate direction. First, the two detection electronic signals must be synchronized inside the AND gate in order to perform the logic operation. These timings can be tuned by programming the time controller, and we check that synchronization by measuring the rate of coincidences between the AND gate output, and the detections in the heralding detector and in Bob's detectors. Second, the wave-packet on Alice's side must pass through the switch when the latter is in the appropriate state. As it takes approximately $\simeq 800\,\text{ns}$ to perform the logic gate and the potential shift of the optical switch's state, we use 300 m-long optical fiber spools, on each party's side, in order to delay the photon for $\simeq 1.5\,\mu\text{s}$. We can then tune the timing of the AND gate's output electronic signal, again by programming the time controller, so that the photon enters the switch right after its state was set. We check the synchronization by running the protocol with Bob's optimal attack, which consists of replacing Bob's detection signal with a continuous electronic signal. The timing is appropriately set when the rate in Alice's verification detector is maximized.

Note that when performing the protocol with honest parties in our conditions (low dark count rate and low double-pair emission probability), then Alice activates her switch only when Bob actually measures the photon, so she cannot measure any photon in her verification detector. This is expected as we tend to minimize the probability of sanctioning an honest Bob, in order to verify the correctness condition. However, this questions the point of using such an optical switch and fast electronics, just to send void on Alice's verification detector. Physically speaking, this seems equivalent to using the exact same setup with no switch, and send all photons to Bob's verification setup. However, we cannot assume Bob to be honest, even when he is. Therefore, it is of major importance that Alice checks that her state actually is projected on the void, in a cryptographic context.

D. Reflectivities

Now we give a recipe to tune the reflectivities in the experiment, to measure them, and we compare these measurements to our theoretical predictions.

When parties are honest, Bob first sets $z = 1/2$ by blocking Alice's signal, and equalizing the detection rates in D_{V_1} and D_{V_2}. This later ensures an optimized interference, and therefore the correctness condition. Then he can tune y such that the detection rate in D_B equals twice the total rate in D_{V_1} and D_{V_2}, which should ensure the fairness condition. Alice then tunes x in order to optimize the interference visibility, which should complete the setting of reflectivities. If v is significantly lower than 1, Alice and Bob might have to perform some mild adjustments on x and y in order to maximize the fairness and correctness.

After performing a protocol with reflectivities x, y, z we can evaluate them by measuring some specific probabilities. We now give the recipe of this procedure, the results of which are shown in Supplementary Figure 4, for protocols with honest parties, and with VOAs simulating different communication distances. First, we force the switch in state $s = 1$ and measure the detection probability in detector D_A. We can then extract x from the following expression:

$$P_{D_A} = x\eta_A^s. \tag{41}$$

Then we measure the detection probability in detector D_B, and extract y from the expression:

$$P_{D_B} = (1-x)y\eta_B^y. \tag{42}$$

Finally, we force the switch in state $s = 0$ and block Bob's side of the interferometer, such that the photon does not interfere on his verification BS, and we extract z from one of these expressions:

$$\begin{aligned} P_{V_1} &= xz\eta_A^{V_1}, \\ P_{V_2} &= x(1-z)\eta_A^{V_2}. \end{aligned} \tag{43}$$

We see in Supplementary Figure 4 that the experimentally measured reflectivities can deviate from the theoretical predictions derived from the efficiency values. The most plausible explanation is that we might not perfectly set the expected reflectivities in each protocol run. This could happen if the fairness $\mathcal{F}$ and the correctness $\mathcal{C}$ are scarcely sensitive to reflectivities around the optimal configuration. Also some undetected errors might have occurred when measuring the efficiencies in Main Text Table 1, because of some undetected fluctuations, or if we did not perfectly set the reflectivities x, y, z to trivial values when performing that measurement.

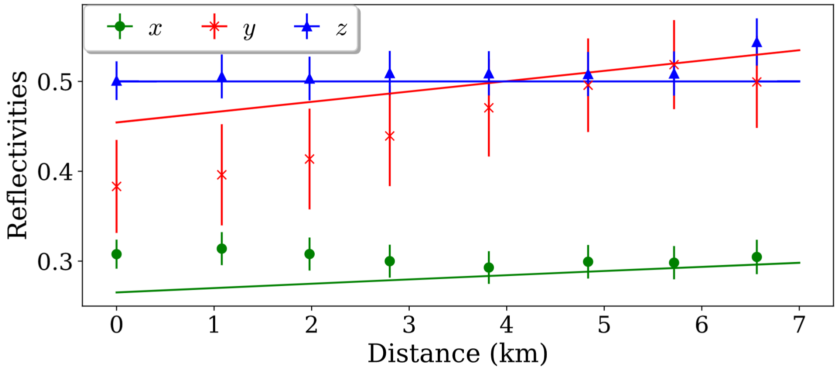

Supplementary Figure 4. Reflectivities measured in protocols with honest parties, for different communications distances simulated with VOAs. The lines show the prediction from Eqs. (5) to (7), with efficiencies given in Main Text Table 1, and with some additional factors $e^{-0.02L}$ induced by VOAs. The error bars are mainly due to error propagation on the efficiencies.

E. Measurement of outcome probabilities

First let us recall the five mutually incompatible protocol outcomes:

– Alice wins when $(b, v_1, v_2) = (0, 1, 0)$,

– Alice is sanctioned if $(b, v_2) = (0, 1)$,

– Bob wins when $(b, a) = (1, 0)$,

– Bob is sanctioned if $(b, a) = (1, 1)$,

– The protocol aborts if $(b, v_1, v_2) = (0, 0, 0)$.

We evaluate the probabilities of these outcomes by measuring the different detection rates and coincidence rates, provided by a simple function of our time tagger. However, the time tagger does not provide a direct way of measuring the rate of an event excluding some other event. For instance, in order to measure the rate of "Bob wins" event, we need to measure the rate of detection in Bob's detector, that did not occur at the same time as a detection in Alice's verification detector. In logical notation, we need the event $b \wedge \neg a$. To calculate such event, we use the fact that for any pair of detection events u, v, we have $u \wedge \neg v = u \wedge \neg(v \wedge u)$ such that the rate $R_{u\backslash v}$ of that event can be calculated as $R_{u\backslash v} = R_u - R_{uv}$, with R_u the rate of detection u and R_{uv} the rate of simultaneous detections u and v. In this way, we can easily deduce the formula for the rates of different outcomes in the protocol, summarized in Supplementary Table 1.

Outcome	a	b	v_1	v_2	Logical	Rate
Alice wins		0	1	0	$\neg b \wedge v_1 \wedge \neg v_2$	$R_{hV_1} - R_{hV_1V_2} - R_{hBV_1} + R_{hBV_1V_2}$
Bob wins	0	1			$b \wedge \neg a$	$R_{hB} - R_{hAB}$
Alice is sanctioned		0		1	$\neg b \wedge v_2$	$R_{hV_2} - R_{hBV_2}$
Bob sanctioned	1	1			$b \wedge a$	R_{hAB}
Abort		0	0	0	$\neg b \wedge \neg v_1 \wedge \neg v_2$	$R_h - \{\textit{Rates of all other outcomes}\}$

Supplementary Table 1. Different protocol events, with the corresponding detection outcomes, logical formula and combination of coincidence rates needed to compute the corresponding probability. The rates subscripts correspond to the detectors which simultaneously trigger, h for the heralding, B for Bob's detector, A for Alice's verification detector, V_1 and V_2 for Bob's verification detectors.

[1] M. Bozzio, U. Chabaud, I. Kerenidis, and E. Diamanti, Phys. Rev. A **102**, 022414 (2020).
[2] K. Fradkin, A. Arie, A. Skliar, and G. Rosenman, Appl. Phys. Lett. **74**, 914 (1999).
[3] F. König and F. N. C. Wong, Appl. Phys. Lett. **84**, 1644 (2004).
[4] R. S. Bennink, Phys. Rev. A **81**, 053805 (2010).
[5] N. Bruno, A. Martin, T. Guerreiro, B. Sanguinetti, and R. T. Thew, Opt. Express **22**, 17246 (2014).
